# The Out-Patient Department

**Published:** 1887-07-02

**Authors:** Andrew Clark


### The Out-Patient Department.

II.—By Mr. Burdett; Miss Twining; Professor Tweedy; Mr. Michelli; Dr. Collier; and Sir Andrew Clark.

(*Continued from p.* 148, *vol. ii.*)

### Mr. Burdett.

I think there is a general agreement upon one point: that there certainly is abuse in the out-patient department. When we come to discuss how much abuse there is, there arises great difference of opinion, because it is very difficult to assess a quantity where you have not all the materials at hand. It is impossible to get exact and precise data. I feel that the initial step to take is to get something like uniform practice at all the hospitals. It does not matter so much what system we adopt, provided that we all agree to adopt the same system.

### Miss Twining.

Speaking as a subscriber, I lament the present condition of things, but I believe it arises not from want of means or lack of generosity. I do think that the question of the out-patient department should be looked into, for it is this which stands in the way of hospital success. If only this were looked into I believe that subscriptions would flow in as desired. Unless the evil is gone to the root, we shall never see the funds flourish.

### Rev. R. J. Simpson.

There is, I believe, a very widespread mistrust among the poor on the subject of charity. They say, "Oh, we are not likely to get the best medicine, etc., if we get it on charity." There is a feeling on the part of the poor that for nothing you get literally nothing, and that what you do not pay for in some form or other is of comparatively little value. I think that a system of small payments, under proper safeguards and regulations, would not only benefit our charities, but would give great confidence to subscribers and to the poor. I believe it would go far to restore something like self-support to our great hospitals, so that they should not be continually dependent upon us for subscriptions. I hope and trust that something will arise out of this movement, and we shall find that the hospitals themselves will flourish, while our wealthy subscribers and poor patients will be better satisfied.

### Dr. Drewitt.

I do not see why the out-patient should not pay 2*s.* 6*d.* per visit. At the Victoria Hospital they pay 1*s.* for a ticket, which lasts a fortnight, and 4*d.* for medicine, including cod-liver oil.

### Professor Tweedy.

The question of hospital management is one of which I claim to have some rather extensive knowledge, having been connected with hospitals during the whole of my professional life, and being on the staff of a hospital in the North for seventeen years, connected with the out-patient department, and for the last few years with the in-patient department, seeing something like 6,000 new cases every year. My experience has probably been larger than many, and, on this question of hospital abuse, the result of my observation is that I do not think there is much abuse. I am connected with two hospitals now: one, University College, and in the other I do not believe there is any abuse. In the course of twelve months I have not seen one person who has been unfitted for hospital treatment. I do not wish to propose any scheme to-night to remedy the abuse which does exist. We have had Dr. Collier's views, Dr. Holmes's, and Mr. Burdett's, and views of other gentlemen, with much of which I agree. It is no use bringing schemes and counter-schemes, but I would suggest that this Association might do a very useful work. I have long been in favour of a Royal Commission to investigate the administration of the hospitals of London, and especially the out-patient department. That Royal Commission is impossible, but this Association might very properly institute an inquiry, and allow the public to be educated. The abuse of hospitals, I am confident, depends upon the ignorance of the public as to what the interests of the hospitals are. Several of the speakers have laid stress upon the necessity for having a large number of out-patients for clinical teaching. My personal experience is entirely the opposite. I do not think we need a large number, and, speaking as a teacher, I think that the best teaching is not done from the rare and extraordinary cases, but from the ordinary cases I meet with in my practice. I should like to press upon this Association the importance of the appointment of some sort of a commission to prepare an initial report.

### Sir Andrew Clark.

I hope that Professor Tweedy will do us the honour of bringing his views before us on a subsequent occasion.

### Mr. Michelli.

I consider Dr. Collier's paper has thrown a great deal of light on a most difficult subject. What Mr. Holmes has said with reference to medical selection is rather new to me. I think that every new case coming to the hospital should be seen by the senior officer. What takes up the time is seeing the old cases; but if the senior officer saw the cases in the first instance, a large number of cases might by that means be selected for clinical purposes, the other cases going to the junior officers. I am an opponent to giving up hospital letters, for I believe they stimulate an interest with a certain class of the public, who give to the hospitals for the simple reason that they get something in return. Besides, a patient is only treated if he is a suitable case, so that the letter does not make any difference in this respect, whilst the person giving it has his satisfaction. With regard to the social status question, I have adopted this plan at my hospital, by putting an intelligent officer in the out-patient department, who from time to time selects patients and sends them up to me. He asks what are their circumstances, etc., and if not satisfactory I investigate them. My experience is that there is very little absolute abuse. I should say that out of the last twenty cases I have investigated I

have found only two ineligible. In one of these two cases I told the applicant that I was sorry I could not ask the medical officer to give her his opinion, when her friend immediately said, "I told you so; they make inquiries at this hospital!"

Dr. Collier,

Replying to the various points raised in the discussion, said: I think I ought to explain, that when I proposed to read this paper I told the then secretary that I should have to base my arguments on the experiments of provincial hospitals. I have some practical experience of three large hospitals, having been resident medical officer, and I am quite sure that in provincial hospitals there is a great deal of real abuse. I brought this question up at the committee meeting last week. In my own out-patient department, within the previous few weeks, I had attending me three or four daughters or wives of farmers, and two or three tradespeople in the neighbouring town. These cases might or might not be suitable cases for charity, but these were cases which should have been challenged and an investigation made. If we allow the relations of country farmers to go to the hospital, how can the country doctor make a living at all if he is only to attend them when they are actually ill? There are one or two points with reference to the remarks made by Mr. Holmes. In the first place, Mr. Holmes seems to think that I have laid too much stress on the wage-limit. I do not wish to lay any stress at all upon the wage-limit. Every case must be decided upon its own merits by the person making the investigation. If there were circumstances of suspicion, the case ought to be reported to a sub-committee, who should make a final decision as to whether it was a proper one. It seems to me that almost every workman who is in the receipt of good wages should first of all become a member of a provident dispensary. By doing so, you establish habits of thrift; but I do not think that those people who are members of provident dispensaries should be obliged to obtain the consent or permission of the provident dispensary officer before they can consult the medical men at the hospital. For instance, if the patient were not getting any great benefit from the dispensary, he should, I think, be allowed to go to the hospital. Another point raised is, that we ought to be very careful not to curtail too much of the out-patient department of our hospitals, because you will diminish to a very great extent the interest in the work of the out-patient physician or surgeon. I may say that, as far as my own work is concerned, I have the opportunity of seeing both the out-patients and the in-patients in the hospital with which I am associated, and I must own that I take a great deal of interest in the out-patient department. We have lately instituted a very good provident dispensary, and my fear is that it will diminish the interest which we feel in the out-patient department. There can be no question that abuse does exist, for we find in some towns as many as a third of the inhabitants to be the recipients of hospital charity.

Sir Andrew Clark.

The subject is of such extreme interest that I would gladly have entered into the discussion, but the hour is now too late. I cannot, however, let the subject pass without a few remarks. I have had thirty-five years' connection with hospitals, and during half of that time I have been connected with the out-patient department, and have taken a very active interest in its work. Now, my experience is akin to the experience of Mr. Tweedy. I do not think that in London (at the London Hospital, at any rate) hospital charity is in any material degree abused. I will mention one instance. Some twenty-five or twenty-six years ago, when this subject was much discussed, one of the persons in the discussion came one day to my hospital, and said, "There, look at that man; don't you think that is a case of abuse?" "Well," I said, "perhaps it is, but let us enquire." We did so. We had this person in, and found he was a clerk earning 35*s*. a week, with a sick wife and sick children. Now, if that man's coming to the hospital was abuse of charity, I do not know what constitutes non-abuse. I could mention many cases—the cases of two or three clergymen, which, from the circumstances they laid before me, I say were more worthy than cases of clergymen who might have had smaller incomes. Some of us seem to be aiming at perfection which is not possible. It is, perhaps, good to aim at perfection, if even we cannot get it, so long as it does not bring about disaster. We had better content ourselves if we saw the good immensely preponderating over the evil. At the London Hospital my experience is that the good of the out-patient department far outweighs the evil, so much so that it is substantially at an end. There are two objects to be held in view in the out-patient work. No doubt the primary object is the relief of sick people; but the other object, which is inseparable from it, is the acquisition and communication of knowledge. Unless the out-patient department is attended by a sufficient number of patients, this end is not attained. I declare that, looking back at my work in the out-patient department of the London Hospital, I consider it to have been as good work as I have done elsewhere. The patients were divided into two classes—the old and the new cases—the latter always receiving the first part of my attention. Some of the new cases were a quarter or perhaps half an hour with me, but the old cases did not require much time. Mr. Holmes would say it was a delusion when I first saw an old case, and said, "Go on"; but it was not so. I knew that, say a case of chronic rheumatism, required continuous treatment with the same medicine, and so it was passed on at once. Mr. Holmes said a very striking thing which I learnt among the out-patients. People who come with chronic disorders of their health owe them, as he has said, to themselves. Hospital doctors should tell patients this truth—that chronic disorders are due to the violation of the laws of health or morality. Turning to the question of the decay and diminution of funds to the hospitals, it is, I own, a lamentable fact. There is, it seems, a new disease among philanthropic people—a very curious and fatal one. It reminds me of cancer. The lip, you know, is composed of a number of little bodies called cells. There is a particular cell in the lip which is a little put out, and, instead of permitting itself to perform its proper function, wants to assert itself, and so it gets irritated and affects others. These cells at last egg each other on, and so spread about the tissues of the lip, till by-and-bye they destroy the lip from which they got their growth. Now the disease which has arisen among us is a kind of philanthropic cancer. One gentleman says this is the way they should treat the people, and another that. The public take alarm, and say that the London hospitals are in a frightful state of corruption. That is the way the subscriptions to the hospitals are getting slacker and slacker, and the poor are injured.

On the motion of Dr. Steavenson, seconded by Mr. A. O'D. Bartholeyns, a hearty vote of thanks was passed to Sir Andrew Clark for presiding.

Mr. Bartholeyns explained the reason that the meeting had not been held at his hospital (the Middlesex). The festival dinner was to have been held upon that evening, and hence it was impossible to fix the meeting there. The dinner was subsequently postponed, and that accounted for his presence that evening. He hoped, however, that The Hospitals Association would be able to meet in his board-room on a future occasion.

The proceedings then terminated.

